# Does the Absence of a Supportive Family Environment Influence the Outcome of a Universal Intervention for the Prevention of Depression?

**DOI:** 10.3390/ijerph110505113

**Published:** 2014-05-13

**Authors:** Susan H. Spence, Michael G. Sawyer, Jeanie Sheffield, George Patton, Lyndal Bond, Brian Graetz, Debra Kay

**Affiliations:** 1 Griffith Health Institute, Griffith University, Nathan Campus, 170 Kessels Road, Nathan, QLD 4111, Australia; 2 Research and Evaluation Unit, Women’s and Children’s Hospital, and Discipline of Paediatrics, University of Adelaide, North Terrace Adelaide, SA 5005, Australia; E-Mail: michael.sawyer@adelaide.edu.au; 3 School of Psychology, University of Queensland, Sir Fred Schonell Dr., St. Lucia, QLD 4072, Australia; E-Mail: jeanie@psy.uq.edu.au; 4 Centre for Adolescent Health, Murdoch Children’s Research Institute, University of Melbourne, 2 Gatehouse Street, Parkville, VIC 3052, Australia; E-Mail: gcpatton@unimelb.edu.au; 5 Centre of Excellence in Intervention and Prevention Science, 15-31 Pelham Street South Carlton, VIC 3053, Australia; E-Mail: lyndalbond@ceips.org.au; 6 *beyondblue*, P.O. Box 6100, Hawthorn West, Melbourne, VIC 3122, Australia; E-Mail: brian.graetz@beyondblue.org.au; 7 International Centre for Allied Health Research, School of Health Sciences, University of South Australia, City East Campus, Cnr of North Terrace and Frome Rd., Adelaide, SA 5000, Australia; E-Mail: debra.kay@internode.on.net

**Keywords:** depression, anxiety, prevention, adolescents, school

## Abstract

To date, universal, school-based interventions have produced limited success in the long-term prevention of depression in young people. This paper examines whether family relationship support moderates the outcomes of a universal, school-based preventive intervention for depression in adolescents. It reports a secondary analysis of data from the *beyondblue* schools research initiative. Twenty-five matched pairs of secondary schools were randomly assigned to an intervention or control condition (*N* = 5633 Grade 8 students). The multi-component, school-based intervention was implemented over a 3-year period, with 2 years of follow-up in Grades 11 and 12. For those available at follow-up, small but significantly greater reductions in depressive and anxiety symptoms and improvements in emotional wellbeing were found over time for the intervention group compared to the control among those who experienced low family relationship support in Grade 8. For those who did not experience low family relationship support in Grade 8, no significant effects of the invention were found over the control condition. This pattern of results was also found for the intent-to-treat sample for measures of depression and anxiety. Previous research may have overlooked important moderating variables that influence the outcome of universal approaches to the prevention of depression. The findings raise issues of the relative costs and benefits of universal *versus* targeted approaches to the prevention of depression.

## 1. Introduction

Most universal interventions designed to prevent depression in young people have focused on the development of resiliency skills. Reviews of the research have typically concluded that the effects are weak and, where significant, are not generally maintained over time [1]. There are several possible reasons to explain these weak results. Interventions have tended to be brief, with sample sizes not large enough to provide sufficient statistical power to detect the relatively small effect sizes expected from universal prevention trials. Furthermore, most interventions have failed to address the environmental factors that are known to impact upon the mental health of young people [2].

In an attempt to overcome these limitations, Sawyer, Pfeiffer, *et al.* [3] developed a multi-component, school-based prevention approach that was implemented over a 3-year period. The intervention reflected a conceptual model of adolescent depression which proposed that, by enhancing psychosocial skills and a protective school environment, it would be possible to break the trajectory between life adversity and depression [4]. The components included a comprehensive classroom curriculum to develop resiliency skills for coping with challenging life situations, enhancements to the school environment, and improvements in pathways to care for those in need of professional assistance. At the end of the 3-year intervention there were no significant differences in depressive symptoms or resiliency skills between students in participating schools and those in control schools [3]. At 2-year follow-up, still no differences were found between conditions on these variables [5]. 

A further explanation for the lack of significant effects in many universal school-based preventive programs may be that such interventions may not be of benefit to all young people and effects may be limited to specific subgroups. It is possible that positive outcomes, in terms of preventing the development of depression, may be greater for those young people who are at particular risk for depression due to the presence of risk factors and/or the absence of protective factors. The focus of most preventive interventions for depression is upon building resiliency skills that assist young people to deal more effectively with life challenges. It is feasible that such interventions are of less benefit to those who live within a protective home environment that provides strong support to assist the young person to deal with life challenges. To date, there has been relatively little research examining the moderators of universal preventive programs for depression. The few studies to date have generally examined factors such as age, gender and initial levels of depression. Generally, there is little evidence to suggest an impact of age and gender upon outcome [3,6,7]. In terms of initial levels of depression the findings are conflicting. While there is some evidence that universal prevention is more effective (at least in the short-term) for those with high baseline levels of depression [6], other studies have not found this effect [3,7]. 

The focus of the present study is upon the role of family support as a moderating variable upon the impact of universal preventive intervention upon depressive symptoms. There are specific reasons for proposing this relationship. For many young people the family environment provides a strong source of support to assist them to deal with life challenges. A proportion of young people, however, live within family contexts that do not provide such support, leaving the adolescent to rely upon their own skills to deal with difficult life events. There is a well-established relationship between low family support and the development of depression, and family support has also been found to buffer the impact of adverse life contexts upon the development of depression [8,9,10,11]. 

We argue that family support is also likely to moderate the impact of universal interventions designed to prevent depression. We propose that interventions that aim to develop resiliency skills will most likely be of benefit to young people who experience low levels of family support and who are thus reliant upon their own skills for handling difficult life situations. Without such intervention, we suggest that young people whose home environment does not provide them with the support needed to assist them to negotiate life challenges will be at increased risk of experiencing high levels of depressive symptoms during their high school years. In contrast, we propose that such interventions will be of less benefit to those who have a supportive family context to assist them to deal with life challenges and for whom the development of resiliency skills is less crucial in determining their mental health outcomes. 

We could identify only one study that has examined the role of family factors in predicting the outcome of a depression prevention program but this was an indicated intervention in which adolescents were selected on the basis of elevated depression scores at baseline [12]. That study did not find that parental support moderated the impact of the preventive intervention upon depressive symptoms, but the findings from an indicated study cannot necessarily be extrapolated to a universal population. Given the established association between low family support and depression, the indicated sample would be more likely to include a greater proportion of young people with low family support than would be the case for a universal sample. 

To summarize, we propose here the level of family relationship support will moderate the impact of a universal, school-based preventive intervention upon depression, anxiety and emotional wellbeing. Specifically, it was hypothesized that young people who, in Grade 8 (G8), experienced a family environment characterized by low levels of support would show significantly greater reductions in depression if they participated in the intervention compared to those who did not. In contrast, it was predicted that there would be no significant difference in mental health outcomes between the intervention and control conditions for young people who did not experience low family support. 

The research question was examined through a secondary analysis of the data from the study reported by Sawyer, Harchak, *et al.* [5]. While secondary analyses of data sets should be treated with caution, given the risk of type 1 errors, the present study can be strongly justified in that the hypotheses are derived from the original theoretical model, outlined above. Petticrew *et al.* [13] make a strong case for sub-group analyses when there is a theoretical rationale behind the hypotheses, and where specific directional effects can be predicted. 

## 2. Methods

### 2.1. Participants

**Figure 1:**
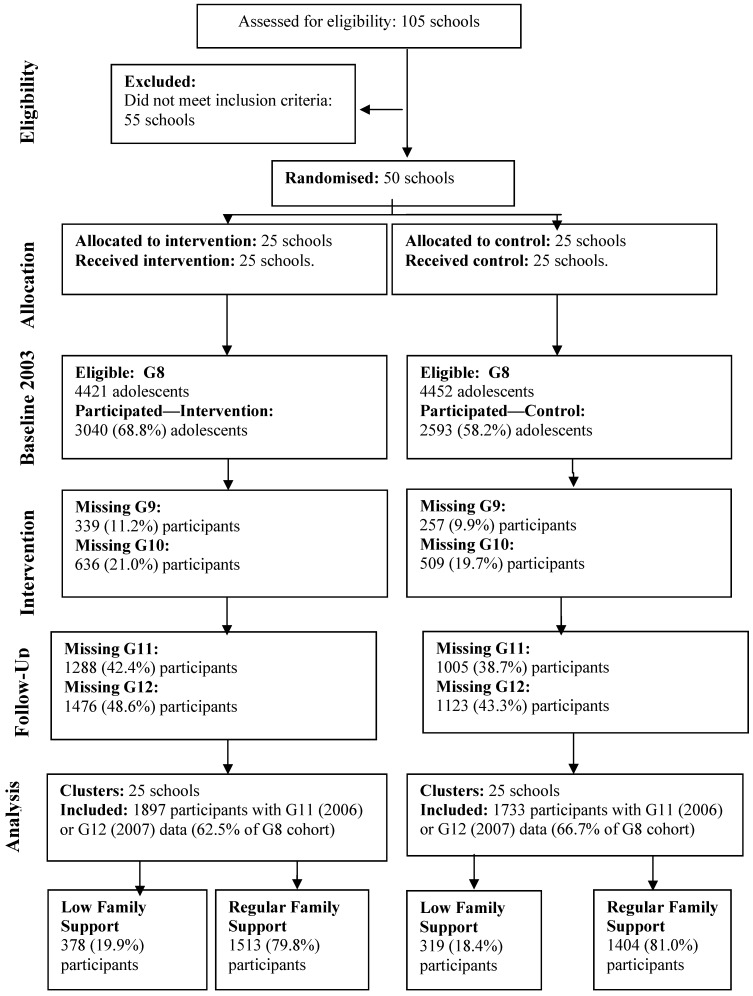
Flow of adolescent participants through study.

Note: G8 = Grade 8; G9 = Grade 9; G10 = Grade 10; G11 = Grade 11; G12 = Grade 12.

Participating adolescents (*N* = 5633) were enrolled in Grade 8 (G8) in 50 secondary schools (25 intervention and 25 control schools) in 2003. Participating schools were located in three Australian States: Queensland (*N* = 18), South Australia (*N* = 16) and Victoria (*N* = 16). To be eligible, schools were required to have an enrolment of at least 100 students in G8 and be willing to participate in either the intervention or control condition. Eligible schools were recruited through an “expression of interest” process. The 105 schools that expressed interest were classified into one of the six following categories: (i) metropolitan government schools ranked in the top third of socioeconomic status (SES); (ii) metropolitan government schools ranked in the middle third of SES; (iii) metropolitan government schools ranked in the lower third of SES; (iv) non-metropolitan government schools; (v) Catholic schools; and (vi) independent schools. For each State, schools within each category were matched as closely as possible in terms of SES and total enrolment size to create ‘school pairs’. Twenty five matched school pairs were identified, with at least one pair from each category. Schools in each pair were then blindly and randomly allocated to either the intervention or control group. The majority of schools were mixed gender (*n* = 42, 84%), government funded (*n* = 34, 68%) and metropolitan (*n* = 38, 76%). Informed consent (parent and student) was provided by 69% of families from the intervention schools and 58% of families from control schools. Figure 1 summarizes participant numbers for each year of the study. 

The 5633 adolescents present at baseline had an average age of 13.08 (*SD* = 0.54) years with 53% being female. Eighty-one percent of participants reported having at least one parent in full time employment, while 70% had parents who lived together (rather than separated, divorced, always single parent, or deceased). Ninety two percent were born in Australia, 14% spoke another language in addition to or rather than English at home and 4.7% identified as being of Aboriginal or Torres Strait Islander background. These figures are consistent with national population estimates for Australia [14].

For the present study, data were analysed for a completer sample of 3630 students who remained enrolled at their original school and completed assessment in G11 and/or G12. These were students in whom the study was specifically interested in as they were present for the three years of the intervention period and continued to attend the school in which the school environment changes had been embedded in the intervention condition. These participants included 1897 adolescents from the intervention schools (62.5% of initial intervention cohort), and 1733 adolescents from control schools (66.7% of initial control cohort). The mean age at the first assessment in G8 was 13.02 (*SD* = 0.53), and 56% were female. 

Eighty-five percent reported having at least one parent in full time employment, while 75% had parents who lived together (rather than separated, divorced, always a single parent, or deceased). Ninety four percent were born in Australia, 12% spoke another language in addition to or rather than English at home and 3.2% identified as being of Aboriginal or Torres Strait Islander background. 

Assessments took place annually from G8-12, providing 5 years of data. Figure 1 indicates the participation rates at each assessment point. There were two main reasons why students were not assessed for the G11 and/or G12 follow-ups. The first was that the ethics approval for the study required that we re-seek written, informed consent from parents and adolescents for the G11 and G12 follow-ups, resulting in an inevitable drop in participation rates. The second reason was that a significant proportion of students in Australia leave school at the end of G10 to enter the workforce, apprenticeship or a technical training course. It was extremely difficult to contact students once they had left the school. To be included in the analyses, students were required to have completed at least three of the 5 annual assessments, one of which was in G8 and at least one in G11 or G12.

The intervention took place over 3 years from G8 to G10, with follow-up over the next 2 years in G11-12. Ethics approval was obtained from the Department of Education and Children’s Services South Australia; Department of Education, Employment and Training Victoria; and Queensland Government Department of Education and the Arts. 

### 2.2. Measures

#### 2.2.1. Center for Epidemiological Studies Depression Scale (CES-D)

The CES-D consists of 20 items regarding frequency of experience of depressive symptoms in the past week [15]. Respondents rate their experience of each symptom on a 4-point scale from “Rarely or none of the time (less than 1 day)” to “Most or all of the time (5–7 days)”. Summed scores can range from 0 to 60 with higher scores indicating more depressive symptoms. The scale has strong reliability and construct validity [15,16]. Internal consistency in the present study was high (Cronbach alpha = 0.90). 

In terms of clinical cut-off points, research has demonstrated that scores >24 are indicative of moderate/severe depression among adolescents [17]. This cut-off has also been used in previous research evaluating universal prevention of depression among adolescents and thus enables comparability of findings with the present study [18].

#### 2.2.2. Anxiety Symptoms

The frequency with which adolescents experienced anxiety was measured using an 8-item scale adapted from the Spence Children’s Anxiety Scale [19]. Participants were asked to indicate how often they experienced each symptom using a 4-point scale (“Never”, “Sometimes”, “Often” or ”Always”). Total scores ranged from 0 to 24, with higher scores reflecting higher levels of anxiety symptoms. Internal consistency was high (Cronbach alpha = 0.89).

#### 2.2.3. Emotional Wellbeing

The degree to which students were satisfied with life and engaged in positive emotional thoughts, acts and feelings was assessed using the 12-item short form of the Mental Health Inventory—Emotional Well-being Scale [20]. Items are rated on a 6-point scale (0 to 5), assessing frequency of occurrence over the past month. Scores range from 0 to 60 with higher scores indicative of greater emotional well-being. Internal consistency in the present study was high (Cronbach alpha = 0.94). 

#### 2.2.4. Supportive Family Relationships

Family relationship support was assessed using five items. The first four items were the family subscale from the Multidimensional Scale of Perceived Social Support (MSPSS) [21] (rated 1 to 7 for level of disagreement/agreement). Examples of items include “I get the emotional support and help I need from my family” and “My family really tries to help me”. The fifth item was generated specifically by the researchers for this study and stated “Sometimes families may have difficulty getting along with one another. They do not always agree and they may get angry. In general, how would you rate your family’s ability to get along with one another?”. This item was rated on a 5-point scale ranging from “poor” to “excellent”.

Total scores ranged from 5 to 33, with high scores representing high family supportive relationships. Factor analysis of the items indicated a single factor with all items loading above 0.63, with an eigenvalue = 3.27, explaining 65% of the variance in scores. Internal consistency of the scale was adequate (Cronbach alpha = 0.79). 

Students were categorized as “experiencing low family relationship support” (LFS) if they reported scores of 22 or below on the 33-point scale (*M* = 26.58, *SD* = 5.07). This cut off was selected as it represented the score approximating 1SD below the mean and included just below 20% of the total baseline sample. There were 697 LFS adolescents (19.3%) of the total sample, with the remaining 2917 students who did not experience low family support being classed as “regular family support” (RFS). The LFS participants included 378 (19.9%) from the intervention group and 319 (18.4%) from the control group, χ^2^ (1) = 1.26, *p* = 0.26. This left 1513 (79.8%) of the intervention condition and 1404 (81.0%) of the control condition categorized as RFS.

### 2.3. Procedure

#### 2.3.1. Intervention

The intervention aimed to strengthen individual cognitive and behavioural competencies, qualities and skills, and enhance supporting factors within the young person’s school environment; [4]. A detailed summary of the program can be found in Spence, Burns, Boucher *et al.* [4] and program materials, including curriculum content, can be downloaded from http://onlinelibrary.wiley.com/doi/10.1111/j.1469-7610.2009.02136.x/suppinfo. All young people attending participating schools during the years of the project took part in the intervention, irrespective of their participation in the research evaluation component of the study. The intervention consisted of four elements, namely:

*Curriculum Intervention*—Psychosocial skills were taught through a classroom program that was delivered sequentially for one term, each year over a 3-year period, from G8 to 10. Each year the program was delivered by teachers through 10 classroom sessions, each lasting 40–45 min, during a single school term. The curriculum was designed to teach a range of cognitive and behavioral skills including: interpersonal problem solving, emotional regulation, social skills, conflict resolution, assertiveness, building social support, adaptive cognitive styles (e.g., increasing positive, but realistic, expectancies and constructive views of the self, the world, and the future), self-efficacy, self-awareness, sense of purpose, and facilitating the understanding of mental health issues and readiness and capacity to seek help for the self and others. 

Each year, teachers completed a 1-day training prior to implementation. Delivery methods included a range of didactic presentations, large and small group exercises, written and oral tasks, workbooks and videotaped examples and dramatized stories that illustrate target skills.

*Building a Supportive School Climate*—this element aimed to build a supportive school environment to promote positive social relationships and safety within the school community, and increase students’ sense of connectedness and belonging in school. The change process in each school was led by a school action team, consisting of several school staff in partnership with a research team facilitator. The school action team developed and implemented an action plan for whole school change, informed by feedback from an audit of school health promotion activities, and student and staff survey data relating to emotional wellbeing and school climate. This component of the intervention was implemented over a 3-year period. Some examples of initiatives included enhancing safety within the school environment, anti-bullying interventions, student peer mentoring, increasing student participation in school governance, and enhancing staff-student communication skills. The facilitator met with the school action team approximately monthly, supported by weekly telephone contact. The first year of the process focused on training the school action team members, conducting an audit of the school’s existing mental health promoting activities, comparing this with good practice principles, and developing the school action plan. Action teams reviewed the implementation, progress, and outcomes of their action plans at six-monthly intervals. Full details of this aspect of the intervention may be downloaded from: http://onlinelibrary.wiley.com/doi/10.1111/j.1469-7610.2009.02136.x/suppinfo.

*Building Pathways for Care and Education*—this component aimed to facilitate adolescents’ access to support and professional services at school and in the community. It provided staff and students with information and resources relating to mental health issues, help and support services, and methods of referral and established clear partnerships between families, school staff, education support/welfare personnel and community-based health professionals. Teachers attended training sessions that examined the roles and responsibilities for educators in relation to mental health promotion, prevention and early intervention. In addition, students developed their own local mental health resource materials. Each school developed a mental health charter and interagency protocols for ensuring students, staff and families could access services in a timely manner.

*Community Forum*—this program element provided young people, their families, and school personnel with information to assist them to identify problems, to seek help for themselves and to help peers. At the end of G8, or early the following year, each school organized a brief community forum, in partnership with local community organizations, which aimed to build community awareness of the nature and prevalence of mental health issues among school students; risk and protective factors; and help-seeking strategies for students, family members and friends. 

#### 2.3.2. Quality of Implementation

Throughout the three-year period, the quality of implementation of the intervention was closely monitored through project facilitator reports. For each component of the intervention project facilitators rated the progress and quality of implementation in each school at the end of each year. The progress of each component was rated as, “Not at all”, “Beginning”, “Developing”, “Consolidating” or “Established/completed”. The quality of implementation for each component was rated as, “Not implemented”, “Limited”, “Adequate”, “Very good” or “Outstanding”. After the third (and final year) of implementation, 100% of schools were rated by the facilitators as having consolidated or completed implementation of the curriculum, 95% for the supportive environment component, 96% for the pathways element and 100% for the community forum. In terms of quality of implementation, facilitators rated 80% of schools as being very good or outstanding for the curriculum component, 84% for supportive environment, 72% for pathways and 92% for the community forum (rated the previous year). Further details regarding quality of implementation can be downloaded from http://onlinelibrary.wiley.com/doi/10.1111/j.1469-7610.2009.02136.x/suppinfo.

#### 2.3.3. Control Condition

The control group participated in the Community Forum component only, with subsequent minimal contact with the researchers other than annual data collection. These schools did not establish school action teams. Data collection took place at the same time points as the intervention schools. 

#### 2.3.4. Statistical Analysis

A multi-level, linear, mixed-model approach, with repeated measures, was used to compare linear trajectories over time as a function of treatment condition, and family relationship support status. Level 1 reflected the within-adolescent change on the outcome variable over time; Level 2 the between-adolescent differences, including family support status; and Level 3 the school-level effect, given that students were nested within schools, and included the intervention condition. The approach analysed trajectories for all individuals in the completer sample, based on the available data points thereby enabling all individuals in the completer sample to be included in the analyses irrespective of missing data. The conditional model contained: (i) a condition variable (coded intervention 1; control group 0); (ii) a time variable (coded 0 to 4); (iii) a family support variable (coded 1 for LFS; 0 for RFS); and (iv) the interaction terms between condition, time, and family support. Analyses were also conducted with gender in the model, but no significant interactions with gender were identified. For the main effects analysis, the sample size of 3630 provided a power of 0.98 to detect an effect size (f) of 0.08 with α = 0.05, based on a repeated measures, mixed effects model. 

Given the problem of missing data in the sample, the data were also analyzed using an intent-to-treat data set that included all 5,633 adolescents who were initially present in G8. Multiple imputation was used to replace missing data using SPSS version 21 to produce multiple complete data sets which were then analyzed using a linear mixed model to enable pooled results to be extracted [22]. The fully conditional specification was used and included all variables used in the analyses and their interactions, in addition to demographic variables. Five imputations were used, with results being pooled as outlined by Schafer [23]. This enabled us to determine whether the results obtained from the completer sample differed from those obtained when drop-outs (with missing values replaced) were included in the analyses. 

## 3. Results

### 3.1. Attrition by Family Relationship Support Status and Condition

Examination of attrition by family support status revealed that, in general, attrition from the study over 5 years was significantly greater in the LFS group (42.1%) compared to the RFS group (33.3%), *x*^2^(1) = 31.03, *p* < 0.001. This finding is not unexpected given that we were predicting an association between LFS and adverse psychosocial outcomes. 

There was also a significant difference in attrition between the intervention (37.5%) and the control group (33.3%), *x*^2^(1) = 11.16, *p* < 0.001). Although this difference must be noted, of key importance to the present study was whether there was a differential rate of attrition between the LFS and RFS groups across intervention and control conditions, to ensure that any associations that may be found between low family support and intervention upon outcome could not simply be attributed to differential rates of attrition. Chi square analyses indicated that although there was a higher rate of dropout among the RFS participants between the intervention (35.4%) and control (31.0%) conditions, *x*^2^(1) = 8.98, *p* = 0.003, there was no significant difference in attrition for the LFS youths between intervention (43.9%) and control (39.9%) conditions, *x*^2^(1) = 1.87, *p* = 0.17. This finding was important in that any differences in outcome subsequently found across interventions for the LFS group could not simply be attributed to differential drop-out rates.

### 3.2. Comparison of Grade 8 Characteristics by Level of Family Support and Condition

We then compared the baseline (G8) characteristics of the intervention *versus* the control group by level of family support among the “completer sample” to confirm equivalence of participants in order to determine whether any differences in outcome could potentially be attributed to initial baseline differences in demographic or mental health variables. Students who were categorized as LFS were significantly more likely than their RFS peers to be female (59.1% *vs.* 40.9%, *x*^2^(1) = 3.12, *p* = 0.04), and were more likely to come from a family in which parents were separated/divorced/deceased (36.5% *vs.* 21.8%, *x*^2^(4) = 68.38, *p* < 0.001, but with no significant difference in age, father unemployment, indigenous background, or country of birth. Compared to RFS students, LFS participants also reported significantly higher baseline levels of depression, *F*(1,3560) = 396.00, *p* < 0.001, η^2^ = 0.10 and anxiety, *F*(1,3560) = 109.15, *p* < 0.001, η^2^ = 0.03, and lower level of emotional wellbeing, *F*(1,3560) = 692.45, *p* < 0.001, η^2^ = 0.16. 

There was also a small but significant baseline difference between conditions for anxiety, *F*(1,3560) = 6.18, *p* = 0.013, η^2^ = 0.002, but not for depression or emotional wellbeing. Table 1 indicates that the intervention group reported slightly higher anxiety scores at baseline than the control group. In terms of condition by level of family support interactions at baseline, there was no significant effect for depression or anxiety, but a significant interaction for emotional wellbeing, *F*(1,3560) = 4.89, *p* = 0.03, η^2^ = 0.001. Table 1 indicates that the LFS participants in the intervention condition showed slightly lower levels of emotional wellbeing at baseline than the LFS control students. Given these significant, albeit extremely small, baseline differences, analyses of intervention effects between conditions were conducted taking baseline depression, anxiety and emotional wellbeing as covariates. As this did not impact upon the results, and to facilitate interpretation, the data are reported here without inclusion of covariates.

**Table 1:** Baseline characteristics by family support status by condition for outcome measures for those available at follow-up.

Variable	Condition	Low Family Support	*M*	*SD*	*N*
Depression	Control	No	11.61	9.45	1385
		Yes	20.44	13.11	312
		Total	13.23	10.78	1697
	Intervention	No	12.25	9.74	1491
		Yes	21.08	13.43	376
		Total	14.03	11.16	1867
	Total	No	11.94	9.61	2876
		Yes	20.79	13.28	688
		Total	13.65	10.98	3564
Anxiety	Control	No	6.62	4.24	1385
		Yes	8.28	5.18	312
		Total	6.92	4.47	1697
	Intervention	No	6.76	4.13	1491
		Yes	9.08	5.77	376
		Total	7.23	4.60	1867
	Total	No	6.69	4.18	2876
		Yes	8.72	5.52	688
		Total	7.08	4.54	3564
Emotional Wellbeing	Control	No	41.77	10.85	1385
		Yes	28.18	12.33	312
		Total	39.27	12.32	1697
	Intervention	No	41.55	10.74	1491
		Yes	30.06	12.99	376
		Total	39.23	12.13	1867
	Total	No	41.65	10.79	2876
		Yes	29.21	12.72	688
		Total	39.25	12.22	3564

### 3.3. Outcomes between Intervention and Control Conditions over Time by Level of Risk

#### 3.3.1. Depressive Symptoms

For the CES-D, there was a significant main effect for family support status, *F*(1,3602.98) = 322.74, *p* < 0.001, but not for condition or time. Table 2 shows that students who experienced low family support in G8 tended to show higher depression scores at each time point than those who did not experience such low family support in G8. Significant interaction effects were found for condition by time, *F*(4,3366.70) = 2.80, *p* = 0.025, family support by time, *F*(4,3367.17) = 15.97, *p* < 0.001, and family support by condition by time, *F*(4,3367.17) = 4.50, *p* = 0.001. The interaction between family support by condition by time remained statistically significant when baseline depression was controlled for using a covariate, *F*(4,3355.45) = 4.68, *p* = 0.001. 

**Table 2:** Estimated marginal mean scores and standard errors from Grades 8 to 12 for depression, anxiety and emotional wellbeing scores by family support status and condition.

Grade	Variable	Grade 8 Low Family Support	Depression		Anxiety		Emotional Wellbeing	
	Condition		Mean	SE	Mean	SE	Mean	SE
Grade 8	Control	No	11.65	0.35	6.61	0.15	41.68	0.38
		Yes	20.46	0.62	8.28	0.26	28.50	0.67
	Intervention	No	12.28	0.34	6.76	0.14	41.51	0.37
		Yes	21.14	0.58	9.08	0.24	30.00	0.62
Grade 9	Control	No	12.78	0.37	6.62	0.15	39.32	0.40
		Yes	19.41	0.67	7.31	0.27	31.53	0.73
	Intervention	No	12.97	0.36	6.38	0.14	40.11	0.39
		Yes	19.88	0.62	7.98	0.24	32.17	0.68
Grade 10	Control	No	13.26	0.39	6.45	0.15	38.85	0.42
		Yes	19.57	0.71	8.12	0.28	31.97	0.76
	Intervention	No	13.49	0.37	6.40	0.15	39.68	0.40
		Yes	19.55	0.65	7.98	0.26	33.55	0.71
Grade 11	Control	No	13.28	0.38	6.49	0.15	38.84	0.41
		Yes	19.48	0.69	8.09	0.28	32.02	0.75
	Intervention	No	13.83	0.37	6.62	0.15	39.26	0.40
		Yes	17.46	0.63	7.31	0.26	34.09	0.69
Grade 12	Control	No	13.03	0.38	6.45	0.15	38.90	0.41
		Yes	19.50	0.71	8.37	0.29	30.88	0.77
	Intervention	No	13.88	0.38	6.49	0.15	39.41	0.41
		Yes	16.51	0.66	6.95	0.26	35.40	0.71

**Figure 2:**
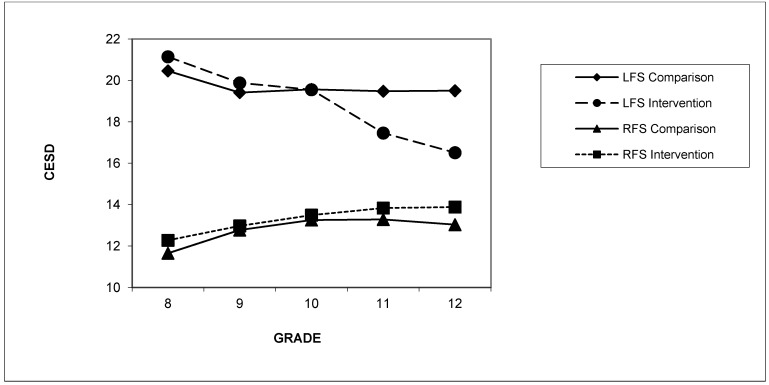
Estimated means for depression scores from Grades 8 to 12 by family support status and condition.

Note: LFS = Low Family Support; RFS = Regular Family Support.

Planned contrasts were conducted to determine the nature of the family support status by condition by time effect between G8-G12. There was a significant condition by time interaction for changes in depression for G8 to G12 for the LFS students, *t*(664.35) = 2.92, *p* = 0.004, *d* = 0.28. The findings are shown graphically in Figure 2. For students categorized as LFS in G8, the intervention condition showed a significant reduction in depression scores of 4.68 points from G8 to G12, *t*(658.39) = −5.43, *p* < 0.001, *d* = 0.28, whereas the LFS students in the control condition showed a small but non-significant increase in depression scores, *t*(669.35) = −0.99, *p* = 0.32, *d* = −0.06. For students categorized as RFS in G8, both the intervention and control conditions showed small but significant increases in depression scores from G8 to G12, *t*(2707.05) = −4.73, *p* < 0.001, *d* = 0.12 and *t*(2659.74) = −3.95, *p* < 0.001, *d* = 0.11, respectively, with no significant interaction between condition and time, *t*(2682.89) = −0.47, *p* = 0.64, *d* = −0.02.

#### 3.3.2. Clinical Impact on Depression

We then examined the percent of participants who exceeded the cut-off for moderate-severe depression, based on CES-D scores >24 for the sample of students who completed assessments in G12 (*N* = 2984). For the RFS sample, there were no significant differences between conditions in G12, χ^2^(1) = 2.16, *p* = 0.14, with 218 (15.4%) of intervention students and 184 (17.6%) of control students exceeding the clinical cut-off point in G12. However, for the LFS sample a significantly lower percentage of participants in the intervention condition exceeded the moderate-severe CES-D cut-off in G12 (*n* = 69, 22.8%) compared to the control group (*n* = 88, 35.2%), χ^2^(1) = 10.41, *p* = 0.001. Analysis of *numbers needed to treat* indicated that approximately 1 in 8 LFS participants would benefit from the intervention in terms of preventing moderate to severe depressive symptoms in G12, 95% CI [5.0, 20.6]. 

#### 3.3.3. Anxiety

Significant main effects were evident for anxiety scores for time, *F*(4,3365.08) = 11.96, *p* < 0.001, and family support levels, *F*(1,3612.031) = 94.28, *p* < 0.001, but not for condition. Compared to RFS participants, the LSF students tended to report higher symptoms of anxiety across all time points. Significant interaction effects were found for family support status by time, *F*(4,3365.46) = 6.89, *p* < 0.001, condition by time, *F*(4,3365.08) = 8.83, *p* ≤ 0.001, and family support status by condition by time *F*(4,3365.46) = 8.66, *p* < 0.001. Estimated mean values are reported in Table 2. The interaction between family support by condition by time remained statistically significant when baseline anxiety was controlled for using a covariate, *F*(4,3355.45) = 8.63, *p* = 0.001

**Figure 3:**
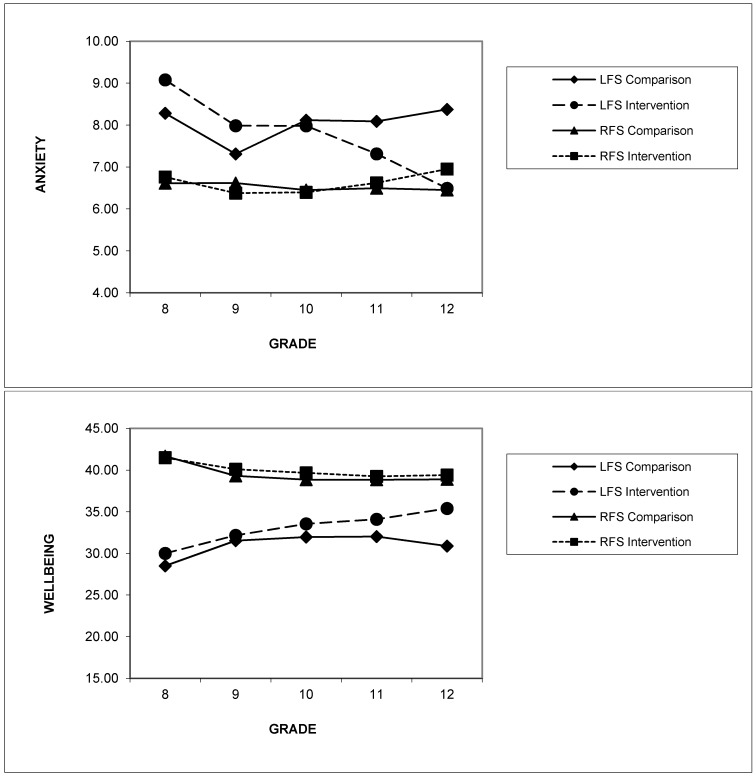
Estimated means for anxiety and emotional wellbeing scores from Grades 8 to 12 by family support status and condition.

Planned contrasts were conducted to examine the interaction effect of family support status by condition by time. The interaction between condition and time for LFS students was significant, *t*(650.02) = 4.58, *p* < 0.001, *d* = −0.41 (see Figure 3). 

For LFS participants, the intervention group showed a significant mean decline in anxiety scores of 2.14 points from G8-12, *t*(643.48) = −6.43, *p* < 0.001, *d* = 0.34. In contrast, LFS adolescents in the control condition showed no significant change in anxiety scores over this period, *t*(655.47) = 0.34, *p* = 0.74, *d* = −0.02. Planned contrasts for the RFS participants indicated no significant interaction between condition by time for changes in anxiety symptoms from G8-12, *t*(2648.19) = 0.56, *p* = *0*.57, *d* = −0.03, with both conditions showing minimal change over time. 

#### 3.3.4. Emotional Wellbeing

Significant main effects were found for family support *F*(1,3600.37) = 427.65, *p* < 0.001, and condition, *F*(1,81.44) = 6.35, *p* = 0.01, but not for time. Participants who reported low family support in G8 tended to report lower levels of emotional wellbeing during the study as shown in Table 2. Significant interaction effects upon emotional wellbeing were found for condition by time, *F*(4,3369.29) = 2.72, *p* = 0.03 and family support by condition by time, *F*(4,3369.29) = 3.17, *p* = 0.01. The interaction between family support by condition by time remained statistically significant when baseline emotional wellbeing was controlled for using a covariate, *F*(4,3355.45) = 3.03, *p* = 0.02.

Planned contrasts between G8 and G12 for the LFS participants demonstrated a significant condition by time effect, *t*(652.08) = −2.31, *p* = 0.02, *d* = −0.24, with the intervention condition showing a significant improvement in emotional wellbeing scores over time, *t*(645.87) = 6.23, *p* < 0.001, *d* = −0.32, (an increase in 5.00 points) and the control condition also showing a significant, albeit smaller, improvement in wellbeing over time, *t*(657.31) = 2.571, *p* = 0.01, *d* = −0.16 (an increase in 2.38 points). Planned contrasts for the RFS participants indicated no significant interaction between condition by time for changes in wellbeing from G8-12, *t*(2978.81) = −1.27, *p* = 0.20, *d* = −0.06, with both the intervention and control conditions showing a small but significant decline in wellbeing scores over time, *t*(2725.82) = −5.78, *p* < 0.001, *d* = 0.15 and *t*(2671.16) = −7.49, *p* < 0.001, *d* = 0.21 respectively. 

#### 3.3.5. Intent-to-Treat Analyses

The analyses were repeated using the imputed data sets for the intent-to-treat sample present at baseline in order to determine whether the results for the completer sample were representative of the full baseline sample, including those who subsequently dropped-out. For depression, the family support by condition by time interaction effect was significant for three of the five imputed data sets, with the pooled results indicating an overall significant effect (*p* = 0.027). For anxiety, there was a significant family support by condition by time effect for all five imputed data sets with a significant overall interaction for the pooled analyses (*p* < 0.001). For emotional wellbeing, the interaction was not statistically significant for any of the imputed data sets, nor for the pooled analyses (*p* = 0.13). Thus, for depression and anxiety, but not emotional wellbeing, the overall pooled results from the imputed samples reinforced those from the completer analyses. 

## 4. Discussion

A strength of the present study is that it included a large enough sample size to provide sufficient power to examine the impact of moderating variables such as family relationship support upon outcome. The analysis of subgroups of participants has not been possible in most previous studies which have typically had much smaller sample sizes. As a consequence, previous evaluations of universal, school-based prevention for depression may have overlooked some important moderating factors. As noted in the introduction, the primary analyses of the present study reported by Sawyer, Harchak, *et al.* [5] found that, for the sample as a whole, there were no significant benefits upon depressive symptoms following a three-year, multilevel, school-based, universal intervention. The secondary analyses conducted in the present study suggest that a different pattern of results is found depending upon the level of family relationship support experienced by the young person in Grade 8. 

It was hypothesized that young people who do not have a supportive family context would be particularly likely to benefit from the school-based intervention as they lack the family support to assist them in tackling the challenging situations of adolescence. Conversely we suggested that, for students who experience a supportive family environment to assist them in dealing with life challenges, the intervention would be of less value. 

Consistent with these hypotheses, we found that those who were categorized as experiencing poor levels of family support in G8 tended to show significantly greater reductions in depressive and anxiety symptoms and improvements in emotional wellbeing from G8 to G12 if they participated in the intervention in contrast to the control condition. The present study also found that the intervention had no significant impact on depression, anxiety, or emotional wellbeing for those participants who experienced more positive levels of family relationship support. Importantly, this pattern of results was consistent across all three outcome measures for the completer sample, and for depression and anxiety in the intent-to-treat analyses using the imputed data sets.

In addition, the results indicated that participants who experienced low family support in G8 tended to show significantly higher baseline levels of depression and anxiety, and lower emotional wellbeing compared to young people with stronger family support. Those with low family support in G8 then tended to continue to show elevated levels of depressive and anxiety symptoms and lower emotional wellbeing through to G12. This suggests that low family relationship support places young people at increased risk for sustained emotional difficulties. Furthermore, participation in the school-based intervention influenced the tendency for mental health problems to persist among those who experienced low family support in G8, with those in the intervention group tending to show greater improvements in anxiety, depression and emotional wellbeing over time than the control group. 

Importantly, the results of the study indicated that the moderating effect of low family support upon the impact of intervention upon mental health outcomes could not simply be attributed to the initially higher levels of emotional distress in G8. When baseline levels of depression, anxiety and emotional wellbeing were included as covariates, intervention effects continued to be significant for those who experienced low family support in G8. 

Despite its strengths, the limitations of the present study must be acknowledged when interpreting the results. In keeping with most studies in this area the sample cannot necessarily be regarded as representative of young people in general. Informed consent to participate in the evaluation could not be obtained from a significant proportion of participants and their parents at commencement of the study. In addition, attrition was relatively high. Those who were not available at follow-up were significantly more likely to have experienced low family support in G8. It is possible therefore, that many of those who were most likely to have benefited from the intervention were not available at follow-up and thus not included in the completer sample. We should note, however, that there was no significant difference in attrition rate for the LFS adolescents across the intervention and control conditions, making it unlikely that the difference in outcomes across conditions for the LFS participants could be attributed to differential dropout rates. Nevertheless, further research, examining the moderating effects of family support upon outcome for universal school-based interventions, is required before definitive conclusions can be drawn.

A further limitation of the present study is that the assessment of mental health was limited to self-report survey instruments and did not include a clinical diagnostic interview. The resources of the study did not enable clinical interviews with such a large sample size, but this should certainly be considered in future research. The use of the CES-D, which is restricted to reports of depressive symptoms over the previous week, also meant that assessment of depression outcomes was limited to specific snap-shots in time and did not enable a survival analysis to be performed. The method by which family support was assessed in the present study also restricts the conclusions that can be drawn. It is possible that youth who exhibit anxiety and depression may have a more negative view of their family support, leading to a bias in reporting. However, low family support in G8 continued to moderate the impact of intervention upon depression when baseline level of depression was included as a covariate, suggesting that the effect is unlikely to simply reflect the impact of high baseline depression levels rather than level of family support. Nevertheless, future studies in this area should include parent report of family support, in addition to the reports of the young person, in order to examine the effects of potential informant bias. Future studies should also examine the impact of family support at each measurement occasion, rather than purely at baseline as was the case in the present study.

Future research should also examine whether other sub-groups of participants are more likely to benefit from interventions of this type. For example, young people who live with a parent with a mental health problem, or who experience abuse or severe adverse life events within the family may also be more likely to benefit from school-based intervention. 

The results of the current study will undoubtedly add to the debate as to whether we should focus our preventive efforts on targeted interventions with specific subgroups rather than universal programs, given the stronger effects found among those who reported family adversity in G8. The effect size for the intervention reported in the present study for the LFS participants was comparable with the overall effect size reported in a recent review and meta-analysis of targeted prevention programs [24]. This same review found no evidence of positive effects upon depression for universal preventive interventions in young people. 

It should be noted, however, that although the results of the present study were statistically significant, the effects were relatively weak from a clinical perspective. In terms of numbers needed to treat, 8 young people would need to take part in the intervention in order to prevent clinical level of depression in one LFS participant. This suggests that the majority of at risk participants, not to mention those with regular levels of family support did not, in general, benefit from the intervention. 

Clearly, research is needed to determine the relative costs and benefits of universal *versus* targeted preventive interventions for depression among at risk young people. It is not straightforward to conclude, based on the findings of the present study, that we should invest in targeted rather than universal approaches to depression prevention given that universal approaches may be of greater benefit to specific subgroups. Targeted approaches may not necessarily be cheaper than universal programs given the economies of scale that can be achieved through classroom, rather than small group, delivery of interventions. Also some elements of school-based interventions, such as whole-school climate change, inevitably require a universal approach and cannot be restricted to specific groups of students. Furthermore, targeted approaches assume that we can reliably identify the subgroups of young people who are most likely to benefit from the interventions. The present study demonstrated that a universal approach can be of sufficient strength to produce a significant preventive effect for a proportion of students from a specific subgroup, namely those with poor family support. Further research comparing universal and targeted approaches and cost-benefit analyses are required before conclusions can be drawn. Such research also needs to take into account the disadvantage of universal approaches that involve young people who are not at risk of developing depression in time-consuming activities that may be of little benefit to them when their time may be better spent on other learning activities. This is an ethical issue that needs to be taken into account in the debate. 

## 5. Conclusions

In conclusion, the study presents some important findings suggesting that prior evaluations of universal approaches to the prevention of depression may have overlooked important moderating variables associated which influence the outcome of universal preventive interventions for depression in youth. It raises issues however, as to the relative costs and benefits of universal *versus* targeted approaches to the prevention of depression in young people. 

## Clinical Trials Registry

This study was initiated prior to the establishment of the registry.
